# A New Species of *Pycnospatha* (Araceae) from Eastern Thailand, with an Updated Key to All Known Species

**DOI:** 10.3390/life16050761

**Published:** 2026-05-01

**Authors:** Wilawan Promprom, Phukphon Munglue, Pattana Pasorn, Soulivanh Lanorsavanh, Wannachai Chatan

**Affiliations:** 1 Department of Biology, Faculty of Science, Mahasarakham University, Kantharawichai District, Maha Sarakham 44150, Thailand; wilawan.pp@msu.ac.th; 2 Plant and Innovation Research Unit, Mahasarakham University, Maha Sarakham 44150, Thailand; phukphon.m@ubru.ac.th (P.M.);; 3 Program of Biology, Faculty of Science, Ubon Ratchathani Rajabhat University, Ubon Ratchathani 34000, Thailand; 4 Walai Rukhavej Botanical Research Institute, Mahasarakham University, Maha Sarakham 44150, Thailand; 5 Department of Biology, Faculty of Natural Sciences, National University of Laos, Vientiane 01080, Laos

**Keywords:** aroid, conservation assessment, dry dipterocarp forest, Indochina, nomenclature, plant diversity

## Abstract

*Pycnospatha* is a small and poorly known genus of Araceae distributed in Indochina and currently comprising only two accepted species. During botanical surveys in Si Sa Ket Province, eastern Thailand, an unusual population of *Pycnospatha* was discovered in a dry dipterocarp forest and found to differ from both *P. arietina* and *P. palmata*. Here, we describe this plant as a new species, *Pycnospatha phanomdongrakensis*. The new species is distinguished by a combination of characters, including a slender habit, shorter petiole and peduncle, a medium-sized spathe, a short and dense spadix, a distinctly curved style directed toward the apex of the spadix, a geophilous and ovoid infructescence, obovate berries, and asymmetrically ovoid seeds. The new taxon is currently known only from a single population in the Phanom Dong Rak mountain range. A preliminary conservation assessment is provided, and the species is treated as Critically Endangered (CR) following IUCN guidelines. An identification key to all species of *Pycnospatha* is also presented. The discovery of this new species highlights the continuing importance of field-based taxonomy in revealing overlooked aroid diversity in the seasonally dry forests of eastern Thailand.

## 1. Introduction

*Pycnospatha* Thorel ex Gagnep. is a small and still poorly known genus of Araceae with a complicated taxonomic history. The genus was first inferred from material collected in Laos by Clovis Thorel during the Mekong expedition of 1866–1868, who recognized the plants as representing an undescribed genus. Nevertheless, this material remained obscure until Gagnepain validly established *Pycnospatha* in 1941 and simultaneously described its two constituent species, *P. palmata* Thorel ex Gagnep. and *P. arietina* Gagnep. In the protologue, Gagnepain compared the genus with *Calla* L. on account of its naked bisexual flowers, but later studies showed that this similarity was superficial and taxonomically misleading. *Pycnospatha* is now correctly regarded as a member of subfamily Lasioideae. The genus is distributed in Indochina, including Cambodia, Laos, Thailand, and Vietnam, and remains narrowly circumscribed, with only two currently accepted species [1,2,3,4,5,6].

Subsequent comparative studies, especially those by Bogner, clarified the systematic placement of *Pycnospatha*. Although morphologically allied to *Dracontium* L., *Pycnospatha* differs in lacking a perigone and in possessing berries with a densely prickly pericarp; its pollen morphology and cytological characters likewise support its placement in subfamily Lasioideae [4,6,7]. The genus is further distinguished by its seasonally dormant, tuberous habit, strongly fornicate spathe, short and dense spadix, bisexual and achlamydeous flowers, and a conspicuously elongate style that extends beyond the stamens.

Species delimitation in *Pycnospatha* has historically been complicated by marked ontogenetic variation in leaf architecture. Juvenile, intermediate, and mature leaves can differ considerably even within a single plant, and such variation has contributed to persistent taxonomic confusion. This is exemplified by *P. arietina*, in which *P. soerensenii* S.Y.Hu was once maintained as distinct, but was subsequently reduced to synonymy when wider morphological evidence demonstrated that the supposed distinguishing characters fall within the developmental range of variation of *P. arietina* [4,5,7,8].

Currently, *Pycnospatha palmata* is known from Laos and has also been reported from Ubon Ratchathani in eastern Thailand, whereas *P. arietina* occurs in Thailand, where it has been recorded from Chanthaburi and Lop Buri, and has also been documented from Cambodia and Vietnam [3,5,6,8,9]. Notwithstanding this progress, *Pycnospatha* remains one of the most poorly collected and least understood genera of mainland Southeast Asian Araceae.

In recent years, the discovery of new species and new distributional records of Araceae in the surrounding region has highlighted the remarkable, yet still incompletely explored, diversity of the family in Thailand. Notable examples include *Amorphophallus coudercii* (Bogner) Bogner, *Amorphophallus sakonnakhonensis* Chatan & Promprom, *Alocasia sakonakhonensis* Chatan & Promprom, and *Colocasia sookchaloemiae* Chatan & Promprom [10,11,12,13]. The discovery and documentation of new aroid species, together with new distributional records from Thailand, indicate that this group is still insufficiently known, with some taxa distinguishable only by subtle morphological or reproductive characters.

Si Sa Ket Province, located in eastern Thailand, has a tropical monsoon climate characterized by a hot dry season, a rainy season influenced by the southwest monsoon as well as occasional tropical depressions and tropical storms, and a relatively cool dry season associated with the northeasterly winter monsoon [14]. During botanical exploration in Si Sa Ket Province, eastern Thailand, we encountered specimens of *Pycnospatha* that could not be assigned to either *P. palmata* or *P. arietina*. Detailed comparison with the protologues, subsequent taxonomic revisions, and recent regional accounts indicates that the Si Sa Ket material represents a distinct and previously undescribed species. The discovery of an additional species in eastern Thailand not only increases the known diversity of *Pycnospatha* but also highlights the continued importance of floristic surveys in the seasonally dry forests of Thailand and adjacent Indochina.

Although molecular phylogenetic data would provide an important independent test of species limits in *Pycnospatha*, the present study evaluates the Si Sa Ket material using detailed comparative morphology of living, cultivated, and herbarium material. Therefore, the objectives of this study are to describe and illustrate the new species, compare its morphology with that of the two previously recognized congeners, and provide a key to the species of *Pycnospatha*.

## 2. Materials and Methods

Material of the putative new species of *Pycnospatha* was collected during botanical surveys in eastern Thailand between 2023 and 2025. Morphological observations were based on living plants in the field, cultivated material, and herbarium specimens deposited at BK and BKF (herbarium acronyms following [15].

Morphological characters were studied from both fresh and dried material, with particular attention given to characters of taxonomic importance in *Pycnospatha*, including leaf form, venation, petiole, spathe shape, spadix structure, floral morphology, style length, and fruit characters. Measurements were made using a vernier caliper, while minute organs were examined and measured under a dissecting microscope equipped with an ocular micrometer. Terminology used in the descriptions follows standard taxonomic practice for Araceae.

For comparative purposes and to facilitate species identification, relevant taxonomic literature, including protologues, taxonomic revisions, and regional floristic treatments, was critically reviewed. For comparative purposes and species identification, relevant taxonomic literature was critically reviewed, including protologues, taxonomic revisions, and regional floristic treatments [2,3,4,5,6,7,8]. Digital images of herbarium specimens, including type specimens of similar species (*Pycnospatha arietina*: *Put 1949* (holotype, K!); *Pycnospatha palmata*: Thorel 2404 (holotype, P!) and other representative collections of allied taxa, were examined through JSTOR Global Plants [16] and individual herbarium websites. These were compared directly with the newly collected material to assess morphological similarity and distinctiveness. No molecular data were included in the present study; accordingly, the taxonomic conclusions are based on comparative morphology.

The preliminary conservation status of the new species was assessed using currently available distributional and ecological data, following the IUCN Red List Categories and Criteria and the recommendations of the [17].

## 3. Results

**Figure 1:**
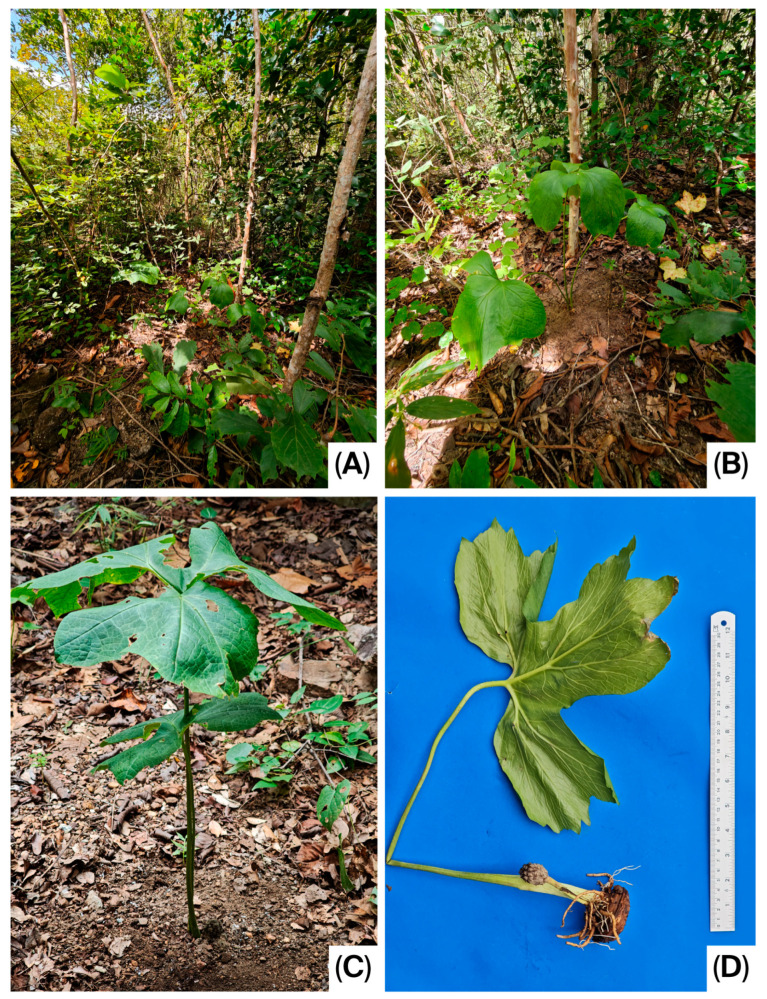
*Pycnospatha phanomdongrakensis*. (**A**,**B**) Habitat; (**C**) Habit; (**D**) Whole plant with infructescence.

**Figure 2:**
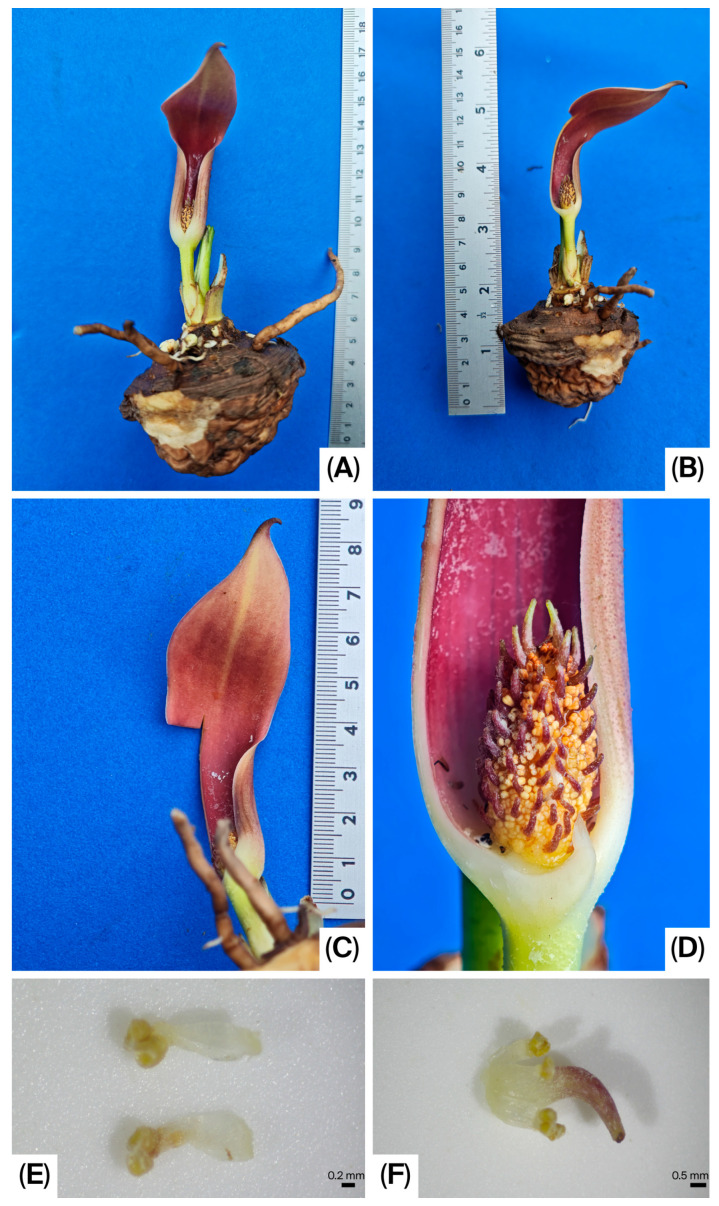
*Pycnospatha phanomdongrakensis*. (**A**,**B**) Tubers, roots and inflorescence; (**C**) spathe; (**D**) Spadix; (**E**) Stamens; (**F**) Androecium and Gynoecium.

**Figure 3:**
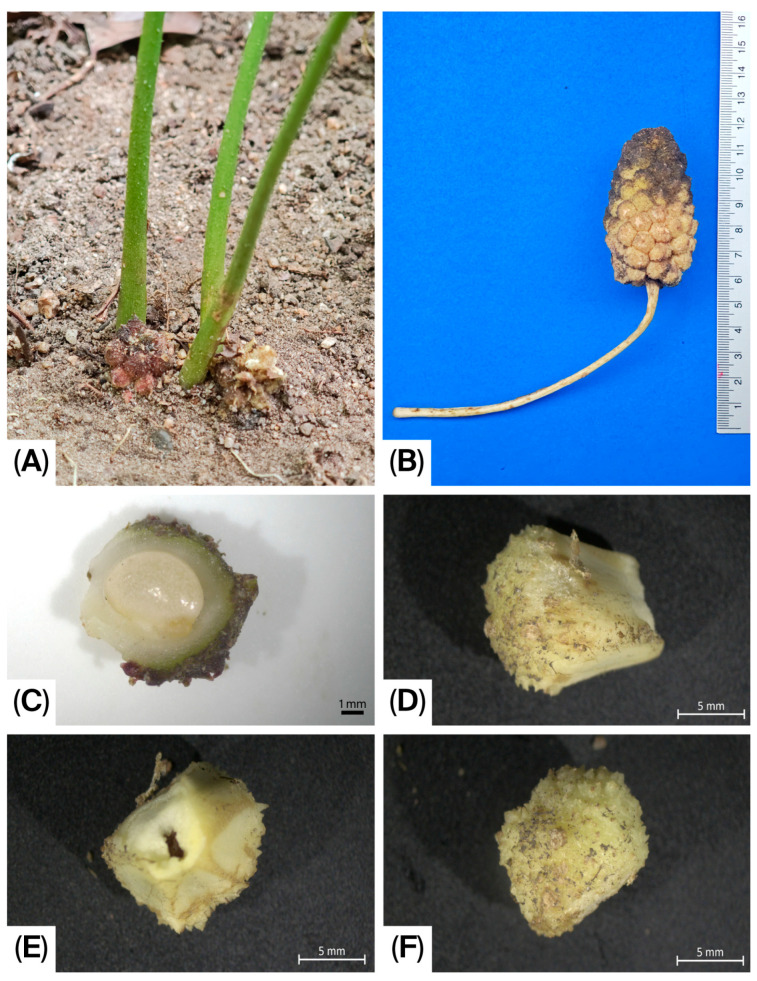
*Pycnospatha phanomdongrakensis*. (**A**) Infructescence mostly below ground; (**B**) Infructescence at the nearly ripe stage; (**C**) Young berry (longitudinal section); (**D**–**F**) Berry at the nearly ripe stage in different views.

**Figure 4:**
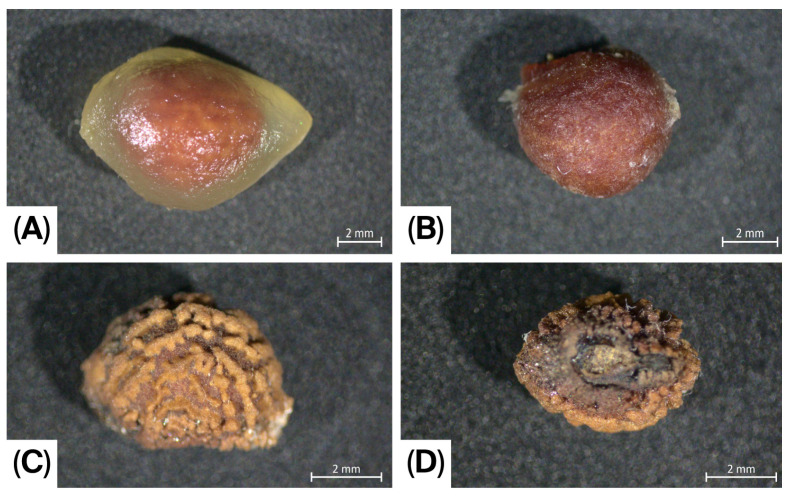
*Pycnospatha phanomdongrakensis*. (**A**) Berry (inner part); (**B**) Fresh seed (lateral view); (**C**,**D**) Dried seed (different views).

**Taxonomic treatment**
***Pycnospatha phanomdongrakensis*** **Chatan & Promprom, sp. nov.**
Figure 1, Figure 2, Figure 3 and Figure 4.

### 3.1. Type

Thailand, Si Sa Ket Provinces. 14°27′22.1″ N 104°33′07.1″ E, 10 August 2025, W. *Chatan 4512* (holotype, BKF!; isotype: BK!).

### 3.2. Diagnosis

The new species differs from *Pycnospatha arietina* in having a much smaller and slender habit (15–60 cm tall vs. robust plants up to 250 cm tall), shorter petioles (15–60 cm long vs. 50–130 cm), smaller spathes (5.5–9 cm long vs. 9–20 cm) and spadices (1.5–1.63 cm long vs. 4–6 cm), and the style distinctly curved and directed toward the apex of the spadix (vs. a straight style). It further differs in having a geophilous and ovoid infructescence (vs. cylindrical or globose), berries obovate, ca. 15.2 × 11.3 mm (vs. globose berries ca. 20 mm in diameter), and asymmetrically ovoid seeds (vs. reniform seeds).

The new species is also distinguishable from *Pycnospatha palmata* by its rough and aculeate petiole, prickles scattered (vs. smooth and not aculeate), typically larger leaf blades that are slightly triangular or hastate to palmatifid (15–25 × 25–35) cm vs. tripartite leaves (to 35 × 16 cm), a medium-sized spathe (5.5–9.0 × 2–3 cm) vs. typically smaller (3–7 × 1.5–3 cm), and larger spadix (1.5–1.63 × 0.6–0.91 cm) vs. considerably more slender (2–3 × ca. 0.5 cm). It further differs by having geophilous and ovoid infructescence (vs. epigeous and cylindrical or globose infructescences); obovate berries ca. 15.2 × 11.3 mm (vs. smaller ovoid or globose berries ca. 7.5 mm in diameter) and asymmetrically ovoid seeds (vs. reniform seeds). Details of the morphological comparison among the new species, *P. arietina*, and *P. palmata* are provided in Table 1.

### 3.3. Description

Geophytic, seasonally dormant, tuberous, slender herb, 15–60 cm tall. Tuber irregularly globose to subglobose or top-shaped; upper surface somewhat irregularly flattened, 3–7 cm in diameter, bearing many roots and many irregular, cylindrical, or globose secondary tubers. Roots stout, 2–4 mm in diameter. Mature leaf solitary at maturity, rarely two together; petiole ca. 15–60 cm long, 1–2.5 cm in diameter at the base, greyish green to whitish grey, with blackish green to reddish or brown mottling; surface rough and aculeate, prickles scattered; subtended by 4–12 × 1–4 cm cataphyll with pale green to whitish surface, brown-spotted; Petiolar sheath short, 1–2 cm long, closed. Mature leaf blade 15–25 × 25–35 cm, slightly triangular or hastate to palmatifid; anterior lobe with one to three lobes, lobe apices acute to acuminate and margins irregularly dentate; posterior lobes one or two lobes, the lobe apices acute to acuminate and margins with irregularly dentate. Leaf maturation acropetal, the leaf blade exposed from the bud at an early stage of expansion; upper surface green and glabrous, lower surface slightly dull green and glabrous. Peduncle 1.8–3.6 cm long, stout, 4–5 mm in diameter, slightly circular in cross-section at the base, ivory at the base and pale green above, enveloped by 1–3 cataphylls. Cataphylls triangular to lanceolate, 11–25 mm long, 10.4–14.2 mm wide, white to ivory at the base, upper part green externally and white internally, with purplish-red margins. Spathe 5.5–9.0 × 2–3 cm; lower part convoluted into a tube, 2.5–5.0 cm long, 1.2–2.0 cm in diameter, margins not overlapping, open, greenish yellow, greenish, or ivory at the base; inner surface of the upper part ivory to orange-red or dark red, outer surface orange-red to dark red. Spathe limb smooth, 2.5–5.0 × 2.0–2.8 cm wide, apex caudate and weakly cucullate, dark red or pinkish orange internally, dark red or grey-green externally. Spadix ca. 0.1 mm stipitate, conical to ovoid, 1.5–1.63 cm long, 6–9.1 mm in diameter, densely covered with flowers, fertile to the apex. Flowers bisexual, achlamydeous, very densely arranged. Stamens 6; filaments 1.0–1.5 mm long, whitish, each with a single longitudinal groove; anthers retuse apically, 1.1–1.3 mm long, strong pale yellow, dehiscing by a longitudinal slit. Gynoecium: ovary ovate, ca. 2.1–2.3 × 1.6–1.75 mm, white to ivory, glabrous; style narrowly conical, 2.5–3.8 mm long, ca. 0.9 mm wide at base, extending well beyond the stamens, the style distinctly curved toward the apex of the spadix; stigma obscure, obtuse; ovule solitary, amphitropous. Infructescence ovoid, rarely elliptic, 6–7 × 3.5–4.0 cm, composed of 30–40 berries, rarely fewer, geophilous. Ripe berries obovate in outline, bearing longitudinal ridge, ca. 15.2 × 11.3 mm, with a persistent style at the apex; pericarp densely covered with conical protuberances or spines, externally strong pale yellow, grey, brown to dark brown, internally strong pale yellow; one-seeded. Seeds asymmetrically ovoid, 5.5–7 × 4.0–4.2 mm; testa hard and thick; dry seeds slightly kidney-shaped, distinctly verrucose, brown; fresh seeds slightly globose, inconspicuously verrucose, brown to dark brown.

### 3.4. Etymology

The specific epithet “phanomdongrakensis” refers to the Phanom Dong Rak mountain range in eastern Thailand, where the new species was discovered.

### 3.5. Phenology

Flowering from February to August; mature fruits present from May to October.

### 3.6. Vernacular Name

Buk Dam.

### 3.7. Distribution and Habitat

**Figure 5:**
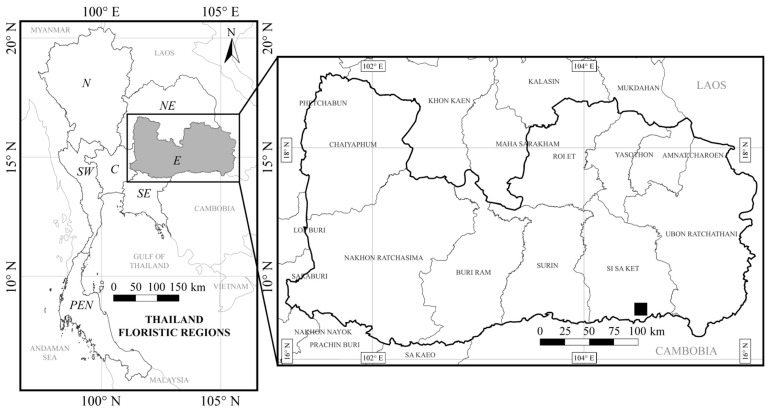
Distribution map of *Pycnospatha phanomdongrakensis* in Si Sa Ket Province, Eastern Thailand (black square).

*Pycnospatha phanomdongrakensis* is currently known from a single population in Si Sa Ket Province, at elevations of ca. 200–250 m. Its distribution is expected to extend to Surin, Buri Ram, and Nakhon Ratchasima provinces, as these areas are located near the type locality and form part of the same mountain range. The species grows under trees in dry dipterocarp forest (Figure 5).

### 3.8. Preliminary Conservation Status

A preliminary conservation assessment based on the IUCN Red List Categories and Criteria [17] indicates that this species is best treated as Critically Endangered (CR) under criterion D, based on its very small population size. At present, only a single population has been documented.

## 4. Discussion

The recognition of *P. phanomdongrakensis* as a distinct species in the current study is supported by a consistent suite of vegetative, floral, and fruit characters that, in combination, fall outside the variation currently documented for the two previously accepted species of the genus, *P. arietina* and *P. palmata*. In the present study, the new species is distinguished by a combination of characters, including its typically smaller and more slender habit, shorter petiole and peduncle, medium-sized spathe, short and dense spadix, distinctly curved style directed toward the apex of the spadix, ovoid infructescence, obovate longitudinally ridged berries with a densely protuberant or spinose pericarp, and asymmetrically ovoid seeds. These characters are stable in the material examined and provide a coherent basis for taxonomic recognition, especially when considered together rather than in isolation. The combination is particularly important in a genus where species have historically been poorly collected and incompletely understood.

Among the two previously known species, *P. phanomdongrakensis* appears morphologically closer to *P. palmata* than to *P. arietina* in several respects, especially in its relatively small stature and the strongly elongated style that is curved toward the spadix apex. However, it differs clearly from *P. palmata* by the rough and aculeate petiole with the scattered prickles, broader and differently shaped mature leaf blades, a generally larger and stouter spathe-spadix complex, and especially by the markedly different fruit and seed morphology. The obovate berries and asymmetrically ovoid seeds of the new species contrast with the smaller berries and reniform seeds reported for *P. palmata*. In contrast, *P. arietina* is a much more robust plant with a substantially larger spathe and spadix, longer peduncle, and a conspicuously straight style; its cylindrical or globose infructescence and globose berries also differ strikingly from the condition seen in the new species. Additionally, the infructescences of *P. phanomdongrakensis* appear to be geophilous, developing below ground with only the apex exposed at the soil surface, whereas those of the other two species are epigeous. These differences support recognition of the Si Sa Ket material as a morphologically distinct taxon within *Pycnospatha*. In total, three species are currently recognized in *Pycnospatha*. The key to all *Pycnospatha* species is provided below.

The taxonomic significance of reproductive characters in *Pycnospatha* deserves emphasis. Previous work has shown that vegetative morphology in the genus, especially leaf architecture, may vary with ontogenetic stage, and that such variation has contributed to taxonomic confusion, as exemplified by the synonymization of *P. soerensenii* under *P. arietina* [4,8]. In this context, the floral and fruiting features documented for *P. phanomdongrakensis* are particularly informative. Characters such as style orientation and length, spadix proportions, infructescence shape, berry morphology, and seed shape are less likely to be explained solely by developmental stage and therefore carry substantial diagnostic value. The present species shows a distinctive reproductive syndrome, notably the strongly curved style, compact spadix, obovate ridged berries, and non-reniform seeds, which together reinforce its separation from previously known congeners.

From a biogeographic perspective, the discovery of *P. phanomdongrakensis* in the Phanom Dong Rak mountain range is significant because it expands the known morphological and likely ecological diversity of *Pycnospatha* in eastern Thailand. Current authoritative treatments continue to regard *Pycnospatha* as a very small Indochinese genus, with *P. arietina* ranging from central Thailand to Vietnam and *P. palmata* recorded from eastern Thailand to Laos [2,3,4,9]. The new species raises the currently recognized species diversity of *Pycnospatha* from two to three, pending future phylogenetic evaluation within this narrowly circumscribed genus, which is restricted to mainland Southeast Asia.

More broadly, the discovery of *P. phanomdongrakensis* highlights the continuing importance of field-based taxonomy in continental Southeast Asia. Even in comparatively well-known monocot families such as Araceae, small seasonal geophytes in deciduous and semi-deciduous habitats remain under-documented. This is especially true for genera with few collections, limited flowering windows, and substantial vegetative plasticity. The new species strengthens the view that the dry-forest regions of eastern Thailand still harbour overlooked aroid diversity and deserve further integrative study. Future work should ideally combine expanded field sampling with comparative anatomy, pollen morphology, and molecular phylogenetic data to test the relationships among the three species of *Pycnospatha* and to evaluate whether the distinctive fruit and gynoecial morphology of *P. phanomdongrakensis* also reflects deeper evolutionary divergence within the genus.

A limitation of the present study is the lack of molecular phylogenetic data, together with the fact that the proposed new taxon is currently known from only a single population. Accordingly, *Pycnospatha phanomdongrakensis* is here treated as a morphologically well-delimited species hypothesis, supported by comparative evidence from living individuals, cultivated material, herbarium specimens, and the relevant taxonomic literature. Nevertheless, additional field collections, broader population-level sampling, and DNA-based phylogenetic analyses will be required to further assess its evolutionary relationships and to test the stability of species boundaries within *Pycnospatha*.

Key to all *Pycnospatha* species

1Style conspicuously straight*P. arietina* Gagnep.1Style distinctly curved toward the apex of the spadix22Petiole rough and aculeate, with scattered prickles, seed asymmetrically ovoid
*P. phanomdongrakensis*
2Petiole smooth and not aculeate, seed reniform
*P. palmata*



## Figures and Tables

**Table 1:** Morphological Comparison of *Pycnospatha phanomdongrakensis* Chatan & Promprom, *P. arietina* Gagnep. and *P. palmata* Gagnep.

Characters	*P. phanomdongrakensis*	*P. arietina* Gagnep.	*P. palmata* Gagnep.
1. Habit	Slender herb with 15–60 cm tall.	Typically, a robust, large herb, up to 2.5 m tall.	A slender herb, up to 70 cm tall.
2. Tuber	irregularly globose to subglobose or top-shaped, with the upper surface somewhat irregularly flattened, 3–7 cm in diameter	irregular globose tuber, 4.5–15 × 2.5–7.5 cm	irregularly globose tuber, 2–5 × 2–2.5 cm
3. Petiole	Medium-sized, 15–60 cm long, 10–25 mm in diameter at the base; surface rough and aculeate, prickles scattered	Typically, 50–130 cm long, 6–10 mm in diameter at the base; surface smooth, rough, or aculeate, the latter with scattered to rather densely arranged prickles.	Small size, 10–70 cm, 2–3 mm at the base; surface smooth and not aculeate
4. Mature leaf blade shape	Slightly triangular or hastate to palmatifid, 15–25 × 25–35 cm	dracontioid, up to 60 × 100 cm,	tripartite, to 35 × 16 cm,
5. Peduncle	1.8–3.6 cm long, 4–5 mm in diameter	8.5–18 cm long, 8–10 mm in diameter, thicker than in related taxa	5–12 cm, 1.5–3 mm in diameter
6. Spathe	Typically, medium-sized, 5.5–9.0 cm long, 2–3 cm wide, weakly cucullate	Typically, larger, 9–20 cm long, 3–5 cm wide, strongly cucullate	Typically, smaller, 3–7 by 1.5–3 cm, strongly cucullate
7. Spadix	1.50–1.63 cm long, 6–9.1 mm in diameter	4–6 cm long, 1–2.3 cm diameter	2–3 cm long, ca 0.5 cm in diameter
8. Stamens	Filaments 1.0–1.5 mm long, anthers 1.1–1.3 mm long	Filaments 1.0–1.2 mm, anthers 2.0–2.5 mm long	Filaments ca. 1 mm, anthers 0.8–1.0 mm long
9. Gynoecium	Style distinctly curved toward the apex of the spadix, 2.5–3.8 mm long; ovary ovate, ca. 2.1–2.3 × 1.6–1.75 mm	Style conspicuously straight, ca. 6 mm long; ovary ovate, 5–7 × 0.7 mm	the style distinctly curved and directed toward the apex of the spadix; ovary ca. 0.8 × 0.35 mm, style 2–4 mm
10. Infructescence	Ovoid, rarely elliptic, geophilous infructescences	Cylindrical or globose, epigeous infructescences	Cylindrical or globose in outline, epigeous infructescences
11. Ripe berries	Obovate in outline, ca. 15.2 × 11.3 mm	Globose, ca. 20 mm diameter	ovoid or globose, ca 7.5 mm diameter
12. Seeds	Asymmetrically ovoid	Reniform	Reniform

## Data Availability

All of the data that support the findings of this study are available in the main text.
